# Antibody Complementarity-Determining Regions (CDRs): A Bridge between Adaptive and Innate Immunity

**DOI:** 10.1371/journal.pone.0008187

**Published:** 2009-12-04

**Authors:** Elena Gabrielli, Eva Pericolini, Elio Cenci, Federica Ortelli, Walter Magliani, Tecla Ciociola, Francesco Bistoni, Stefania Conti, Anna Vecchiarelli, Luciano Polonelli

**Affiliations:** 1 Microbiology Section, Department of Experimental Medicine and Biochemical Sciences, University of Perugia, Perugia, Italy; 2 Microbiology Section, Department of Pathology and Laboratory Medicine, University of Parma, Parma, Italy; Louisiana State University, United States of America

## Abstract

**Background:**

It has been documented that, independently from the specificity of the native antibody (Ab) for a given antigen (Ag), complementarity determining regions (CDR)-related peptides may display differential antimicrobial, antiviral and antitumor activities.

**Methodology/Principal Findings:**

In this study we demonstrate that a synthetic peptide with sequence identical to V_H_CDR3 of a mouse monoclonal Ab (mAb) specific for difucosyl human blood group A is easily taken up by macrophages with subsequent stimulation of: i) proinflammatory cytokine production; ii) PI3K-Akt pathway and iii) TLR-4 expression. Significantly, V_H_CDR3 exerts therapeutic effect against systemic candidiasis without possessing direct candidacidal properties.

**Conclusions/Significance:**

These results open a new scenario about the possibility that, beyond the half life of immunoglobulins, Ab fragments may effectively influence the antiinfective cellular immune response in a way reminiscent of regulatory peptides of innate immunity.

## Introduction

Antibodies (Abs) are formed by heavy and light chains composed of constant and variable regions. The latter include six complementarity determining regions (CDRs) which constitute the antigen (Ag) binding-site. The structural repertoires and the relationships between amino acid sequences and tertiary structures have been extensively studied to reveal the importance of the typical loops, which are canonical structures in the three CDR segments belonging to the light chain (Ll, L2, and L3) as well as the first two CDR segments of the heavy chain (Hl and H2) [1]. The third CDR of the heavy chain (H3) displays wide variety in its length and amino acid sequence, and no canonical structures have ever been established for it [1], [2], [3]. Variety of CDR1 and CDR2 is encoded by the germline and furtherly diversified by somatic mutation while the one of CDR L3 and CDR H3 is somatically generated by rearrangement of the variable (V) segment with the joining (J) L or diversity (D) H and JH segments, respectively. Notably, CDR H3 plays a crucial role in mediating individual Ag recognition, sometimes by changing its conformation upon Ag binding [4], although the other five CDRs are also more or less implicated in increasing binding affinity to Ag and some contact residues can even be situated within framework of variable regions [5].

The observation that Ab specificity is determined by a limited number of residues has prompted the synthesis of small peptides based on CDR sequences which retain binding properties and functions of the intact Ab [6], [7].

In previous studies it has been demonstrated that the CDRs, or related peptidic fragments, of a recombinant single chain Ab (scFv), representing the internal image of a wide antimicrobial spectrum *Pichia anomala* killer toxin (KT), may exert a specific microbicidal activity *in vitro* against KT-sensitive microorganisms characterized by specific cell-wall receptors mainly constituted by 1,3-β-glucans [8]. In particular, a decapeptide related to the CDR L1 of KT-scFv (P6), selected for its relevant *in vitro* candidacidal activity, has been analyzed by alanine substitution (alanine scanning) in order to evaluate the functional contribution of each residue. One of its derivatives (KP), characterized by a significant increase of the candidacidal activity, proved to be active, *in vitro*, against diverse eukaryotic and prokaryotic microorganisms and to inhibit *in vitro*, *ex vivo* and/or *in vivo* HIV-1 and Influenza A virus replication by different mechanisms of action [8], [9], [10], [11], [12], [13], [14], [15]. KP was able to exert a very effective therapeutic activity in experimental models of vaginal and systemic candidiasis, disseminated cryptococcosis and paracoccidioidomycosis as well as Influenza A virus infection [8], [9], [10]. KP proved, moreover, to modulate the expression of costimulatory and MHC molecules on murine dendritic cells (DC), after selective binding, and to improve their capacity to induce lymphocyte proliferation [16]. Recent studies on the structure-function relationship of KP showed its reversible self-assembly in an hydrogel-like state. Significantly, this process is catalyzed by 1,3-β-glucans. KP self-assembled state may provide protection against proteases and regulate the release of the active form over time, while the β-glucans affinity is responsible for targeted delivery [17].

Polonelli *et al.* studied synthetic peptides with sequences identical to CDRs of the light and heavy chain of three monoclonal Abs (mAbs) characterized by different specificity: mAb C7, directed to a protein epitope of a *Candida albicans* (*C. albicans*) cell wall stress mannoprotein, mAb pc42, directed to a synthetic peptide containing well-characterized B-cell and T-cell epitopes, and a human mAb HuA, directed to blood group A substance [18], [19], [20], [21]. The study showed that, irrespective of the specificity of the native Ab, the synthetic CDRs may exert *in vitro, ex vivo* and/or *in vivo* differential inhibitory activities against *C. albicans*, HIV-1 and B16F10-Nex2 melanoma cells, conceivably mediated by different mechanisms of action. Alanine substituted synthetic CDRs, used as surrogates of natural point mutations, showed further differential increased/unaltered/decreased antimicrobial, antiviral and/or antitumor activities [22].

As bioactive molecules, CDR-related peptides may present some advantages over whole Abs of adaptive immunity owing to their small size, e.g. lack of immunogenicity and better tissue penetration, as well as over natural peptides of innate immunity (e.g. defensins, cathelicidins, hystatins) owing to higher specificity and affinity for targets and low systemic toxicity [23], [24].

In the aim to establish whether CDRs may drive a protective cellular immune response exclusively due to their immunomodulatory activity, we studied the synthetic CDRs of mAb HuA and a mouse mAb (MoA) which binds to the same carbohydrate epitope [21], [25]. MAbs MoA and HuA are nearly identical immunochemically, even though present unrelated primary sequences, and are representative of different ways by which the same epitope can be recognized by the immune system [21].

## Results

### Candidacidal and Immunomodulatory Effects of the CDRs Peptides

In recent studies the therapeutic effect of Ab-derived peptides characterized by immunomodulatory and/or direct candidacidal activity has been demonstrated in the experimental model of systemic candidiasis [8], [16], [22].

Here we investigated the immunomodulatory effects of the CDRs of a human IgM mAb (HuA) specific for difucosyl human blood group A substance, previously evaluated for their candidacidal properties [22], as well as the candidacidal and immunomodulatory effects of the CDRs of a mouse IgM mAb (MoA) which bind to the same carbohydrate epitope [21].

First, we tested the capacity of synthetic peptides representing mAb MoA CDRs to kill *in vitro C. albicans* cells. None of them proved to display any candidacidal effect *in vitro* in the adopted experimental conditions.

**Figure 1:**
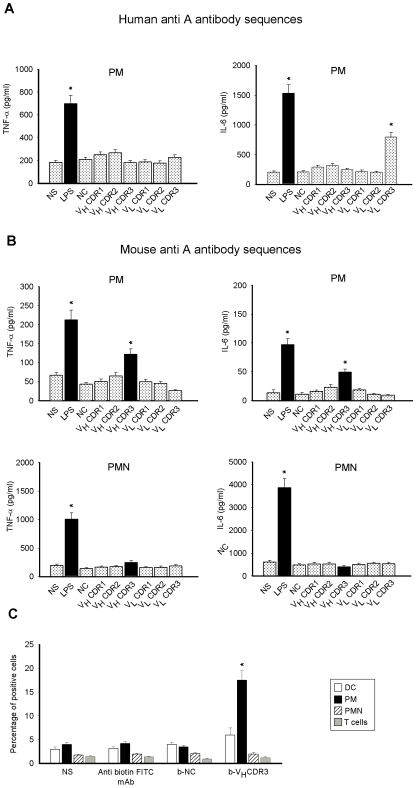
TNF-α and IL-6 production by PM and PMN stimulated with human and/or mouse CDRs and mouse V_H_CDR3 uptake by different cell populations. PM (**A**) or PM and PMN (**B**) (both 5×10^6^/ml) were cultured in the presence or absence (NS) of human and/or mouse CDRs, LPS, or NC (all 10 µg/ml) for 18 h. After incubation, TNF-α and IL-6 levels were evaluated in culture supernatants by specific ELISA assays. *, *P*<0.05 (treated *vs* untreated cells, n = 7). DC, PM, PMN, and T cells (all 1×10^6^/ml) were incubated for 1 h in the presence or absence (NS) of b-V_H_CDR3 or b-NC (both 10 µg/ml). After incubation, permeabilized cells were reacted with FITC-labelled mAb to biotin and analyzed by FACScan flow cytometry. Data are reported as the percentage of positive cells (**C**). *, *P*<0.05 (b-V_H_CDR3 treated *vs* untreated cells, n = 5). Error bars, s.e.m.

In parallel experiments we analyzed the capacity of all murine and human synthetic CDRs to stimulate cytokine production by a non homogeneous cell population such as murine splenocytes. An irrelevant synthetic peptide previously recognized unable to stimulate immune cells was used as negative control (NC) in this set of experiments [16]. Our results showed that mAb HuA V_L_CDR3 induced a significant up-regulation of IL-6 production but not of TNF-α, while mAb MoA V_H_CDR3 was able to induce an increased production of both IL-6 and TNF-α (not shown). Other CDRs or NC did not affect cytokine production by splenocytes. Given that the secretion of proinflammatory cytokines is generally considered a prerequisite of innate immune cells [26], we stimulated purified peritoneal murine macrophages (PM) with all murine and human synthetic CDR peptides. Results showed that, as observed in splenocytes, the stimulation with mAb HuA V_L_CDR3 induced a significant up-regulation of IL-6 production by PM (Fig. 1A), while mAb MoA V_H_CDR3 induced a significant up-regulation of both TNF-α and IL-6 production (Fig. 1B, upper panel). Also in this case other CDRs or NC did not stimulate cytokine production by PM. Subsequently, we also tested the capacity of the murine synthetic CDRs to induce TNF-α and IL-6 production by peritoneal murine neutrophils (PMN). None of murine CDRs stimulated cytokine production after 18 h (Fig. 1B, lower panel). In our experimental system, LPS was used as positive control.

Since mAb MoA V_H_CDR3 showed the better capability to modulate proinflammatory cytokines by PM, with respect to mAb HuA V_L_CDR3, we used this peptide in subsequent experiments. Noteworthy only the V_H_CDR3 alanine-substituted at position 5 derivative resulted in a loss of TNF-α production capacity (data not shown).

### V_H_CDR3 Uptake by Immune Cells

In order to analyze the possible interaction of V_H_CDR3 with immune cells, DC, PM, PMN or T cells were incubated with biotin-labelled (b-) V_H_CDR3 (b-V_H_CDR3) for 1 h and then cell uptake of peptide was determined. The results showed that the peptide receptive cells were PM; conversely, DC, PMN and T cells did not show any significant interaction with b-V_H_CDR3 (Fig. 1C). Binding of NC resulted marginal in each cell population evaluated (Fig. 1C).

**Figure 2:**
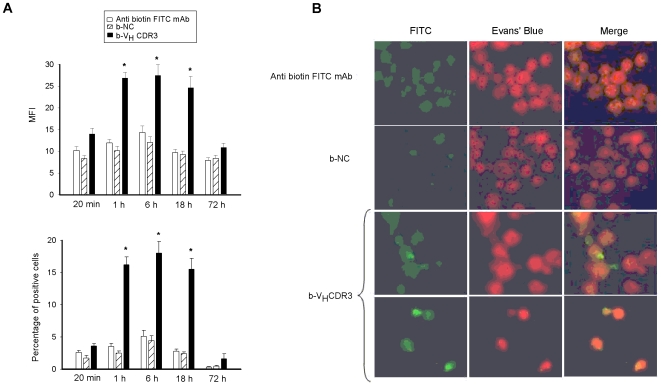
Kinetic of V_H_CDR3 uptake by PM. PM (1×10^6^/ml) were incubated for different times with b-V_H_CDR3 or b-NC (all 10 µg/ml). After incubation, permeabilized cells were reacted with FITC-labelled mAb to biotin and analyzed by FACScan flow cytometry. Data are reported as the mean fluorescence intensity (MFI) (upper panel) and percentage of positive cells (lower panel) (**A**). *, *P*<0.05 (b-V_H_CDR3-treated *vs* untreated cells, n = 7). Error bars, s.e.m. In selected experiments cells were incubated for 1 h as above described, reacted with FITC-labelled mAb to biotin in the presence of Evans' Blue as a counter stain, and subsequently examined under fluorescent light microscopy. Note the green fluorescence of b-V_H_CDR3 treated cells. Original magnification 20×(**B**). Images shown are from one representative experiment out of five with similar results.

Given that V_H_CDR3 stimulates TNF-α and IL-6 production and that significantly binds PM, a physical interaction between macrophages and peptide could be postulated. As a consequence, we analyzed the kinetics (20 min, 1, 6, 18 and 72 h) of V_H_CDR3 uptake by PM. The results showed that V_H_CDR3 uptake was at high level after 1 h of incubation, persisted for 6 and 18 h, and decreased to negligible levels after 72 h (Fig. 2A). Prolonged incubation probably results in degradation or ejection of V_H_CDR3 by the cells, as documented by the rapid decrease of MFI and percentage of positive cells at 72 h (Fig. 2A). Furthermore, V_H_CDR3 uptake was confirmed by fluorescence microscopic analysis (Fig. 2B). A negligible binding of NC was observed at all times tested.

### Activation of Akt Pathway by V_H_CDR3 in PM

**Figure 3:**
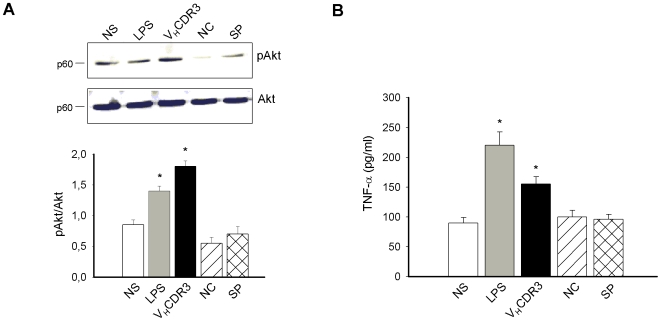
Phospho-Akt activation and TNF-α production in PM stimulated with V_H_CDR3. PM (3×10^6^/ml) were stimulated for 1 h in the presence or absence (NS) of V_H_CDR3, LPS, NC or SP (all at 10 µg/ml). After incubation, cell lysates were subjected to Western blotting. Membranes were incubated with Abs to pAkt and Akt; pAkt was normalized against Akt (**A**) *, *P*<0.05 (treated *vs* untreated cells, n = 5). PM (5×10^6^/ml) were stimulated for 18 h as above described. After incubation, TNF-α level was evaluated in culture supernatants by specific ELISA assays. (**B**) *, *P*<0.05 (treated *vs* untreated cells, n = 5).

The PI3K-Akt network mediates intracellular signals to regulate a variety of cellular responses, including cellular activation, protein synthesis, cell cycling, and survival [27]. Given that PI3K represents a signal transduction event associated with cell activation [28], we considered the possibility that V_H_CDR3-mediated activation might imply PI3K recruitment. Since recent evidence indicates that PI3K-dependent activation of Akt is synonymous of increased PI3K activity [28], we evaluated Akt phosphorylation in cells stimulated for 1 h with V_H_CDR3. As additional control of specific immunomodulatory activity of V_H_CDR3 a scramble peptide (SP) was used. Results showed an increased activation of pAkt in cells stimulated with V_H_CDR3 with respect to stimulation with NC and SP (Fig. 3A). This effect was correlated with increase of TNF-α production (Fig. 3B).

**Figure 4:**
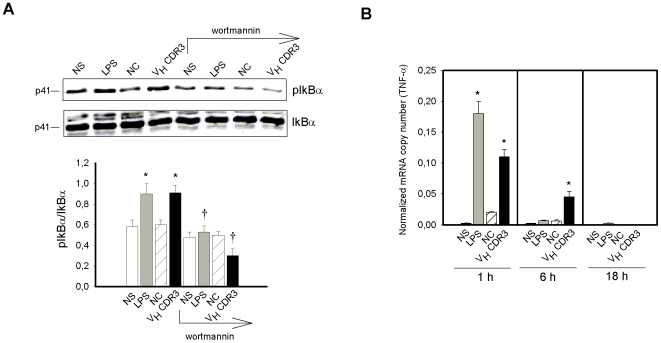
Phospho-IkBα activation and TNF-α gene expression in PM stimulated with V_H_CDR3. PM (3×10^6^/ml) were stimulated for 1 h in the presence or absence (NS) of wortmannin (4 nM), V_H_CDR3, LPS or NC (all at 10 µg/ml). After incubation, cell lysates were subjected to Western blotting. Membranes were incubated with Abs to pIkBα and IkBα; pIkBα was normalized against IkBα. (**A**) *, *P*<0.05 (treated *vs* untreated cells, n = 5); †, *P*<0.05 (wortmannin-treated *vs* wortmannin-untreated cells, n = 5). For testing the expression level of TNF-α gene, PM (1×10^6^/ml) were cultured for 1, 6 and 18 h as above described. After incubation, total RNA was isolated and analyzed for mRNA expression with RT-PCR. Transcript copy numbers were determined by qPCR using cDNA as a template. Copy numbers were normalized against the copy number of the GADPH gene (**B**). *, *P*<0.05 (treated *vs* untreated cells, n = 5). Error bars, s.e.m.

Based on the finding that Akt has been implicated in regulating TNF-α gene activation [27], we analyzed whether such activation occurs via phosphorylation of IkBα, which allows the translocation of NFkB into the nucleus [29]. Results, reported in Figure 4A, showed an increased activation of pIkBα in PM after 1 h of stimulation with V_H_CDR3 as compared to stimulation with NC. Moreover, treatment with wortmannin, a PI3K-Akt pathway inhibitor [30], significantly reduced the V_H_CDR3-induced IkBα phosphorylation after 1 h stimulation (Fig. 4A). This was related to the significant increase in TNF-α gene expression observed 1 and 6 h post stimulation with V_H_CDR3. Prolonged incubation (18 h) resulted in a rapid decline of gene expression. The stimulatory activity of V_H_CDR3 was confirmed by the inability of NC to activate cells (Fig. 4B). Given that p38 MAPK seems to be related to TNF-α production and signaling via PI3K activation, we used either a p38 MAPK inhibitor (SB203580) or wortmannin to evaluate the involvement of p38 MAPK and/or PI3K on TNF-α production. Our results evidenced that treatment with wortmannin reduced TNF-α production induced by LPS and V_H_CDR3 by 39% and 28% respectively. Furthermore, treatment with p38 MAPK inhibitor reduced TNF-α production induced by LPS and V_H_CDR3 by 28% and 21%, respectively.

### Modulation of TLR-4 Expression by V_H_CDR3 in PM

**Figure 5:**
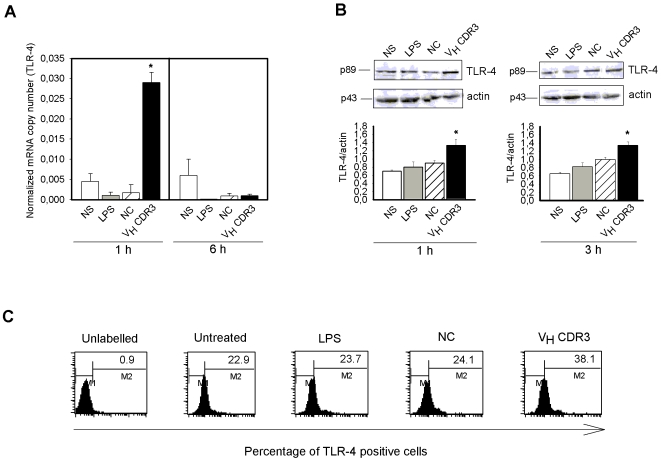
Expression of TLR-4 in PM stimulated with V_H_CDR3. PM (1×10^6^/ml) were cultured for 1 and 6 h in the presence or absence (NS) of V_H_CDR3, LPS or NC (all 10 µg/ml). After incubation, total RNA was isolated and analyzed for mRNA expression with RT-PCR. Transcript copy numbers were determined by qPCR using cDNA as a template. Copy numbers were normalized against the copy number of the GADPH gene (**A**). *, *P*<0.05 (V_H_CDR3 treated *vs* untreated cells, n = 5). PM (3×10^6^/ml) were cultured for 1 and 3 h as above described. After incubation, cell lysates were subjected to Western blotting. Membranes were incubated with Abs to TLR-4 and actin. TLR-4 production was normalized against actin (**B**). *, *P*<0.05 (V_H_CDR3-treated *vs* untreated cells, n = 5). Error bars, s.e.m. PM (1×10^6^/ml) were cultured for 1 h as above described. After incubation, permeabilized cells were reacted with RPE-labelled mAb to TLR-4 and analyzed by FACScan flow cytometry. Values represent the percentage of positive cells (**C**). Data shown are from a representative experiment out of five with similar results.

Convincing arguments point to TNF-α as a potent TLR-4 ligand possibly able to influence TLR-4 expression as well [31]. Since TNF-α is abundantly secreted by PM after stimulation with V_H_CDR3, we analyzed whether TLR-4 expression was regulated in PM in our experimental system. Results showed that there was a significant increase in TLR-4 gene expression in cells stimulated with V_H_CDR3 for 1 h, which declined after 6 h (Fig. 5A). This activation was correlated with a significant up-regulation of TLR-4 protein as evidenced by Western blot analysis 1 and 3 h after V_H_CDR3 stimulation (Fig. 5B). Consistent with these results, a significant increase in the percentage of TLR-4 positive cells after 1 h of stimulation with V_H_CDR3 was observed (Fig. 5C). No activation was observed after stimulation with LPS [32] or NC. This could be due to the capacity of LPS to induce the production of other cytokines such as IL-10 [33] that can downregulate TLR-4 expression [34].

### Influence of TNF-α Production, V_H_CDR3 Induced, in TLR-4 Upregulation in PM

**Figure 6:**
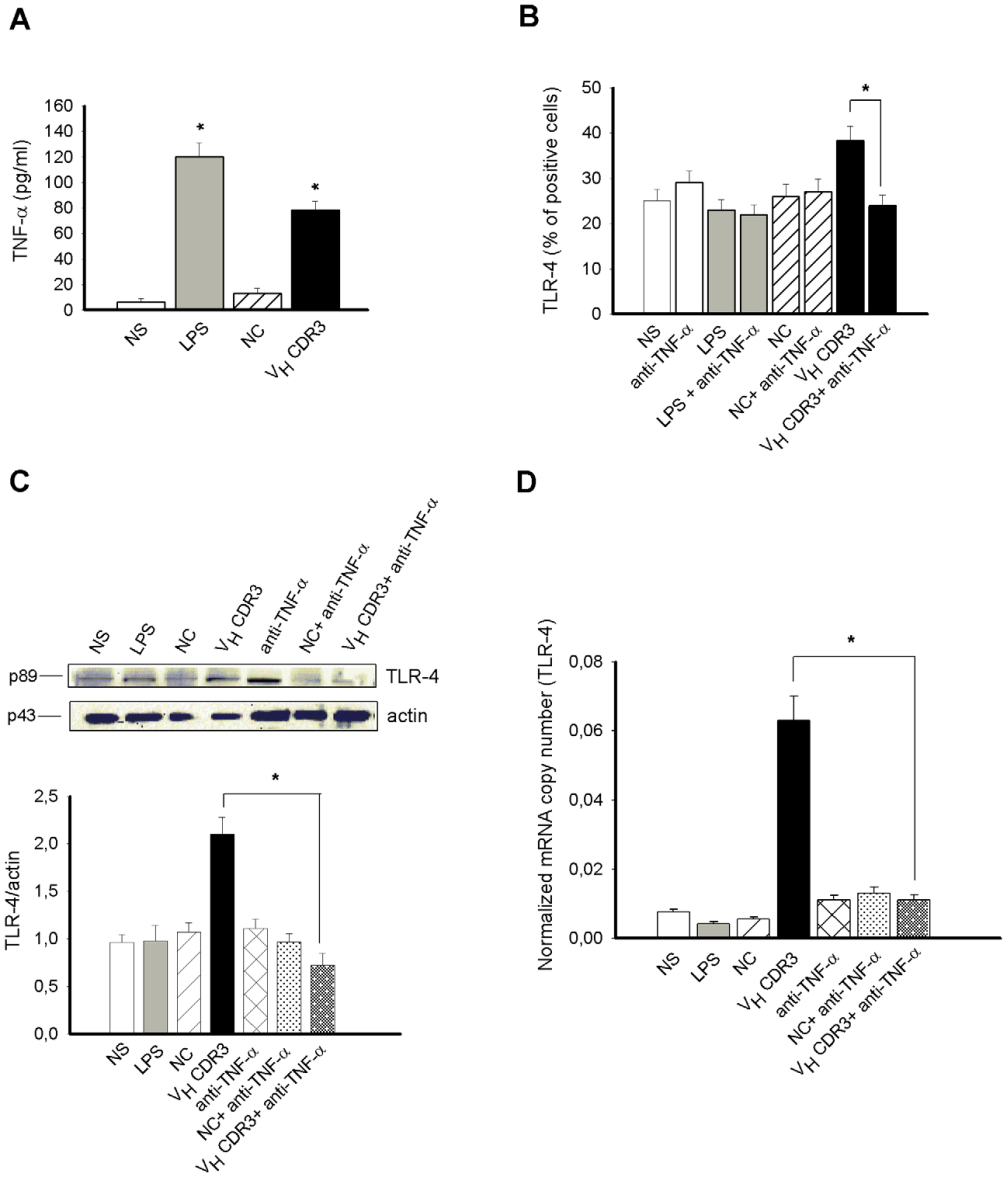
TNF-α induced TLR-4 expression in PM stimulated with V_H_CDR3. PM (5×10^6^/ml) were cultured for 1 h in the presence or absence (NS) of V_H_CDR3, LPS or NC (all 10 µg/ml). After incubation, TNF-α level was evaluated in culture supernatants by specific ELISA assay (**A**). *, *P*<0.05 (treated *vs* untreated cells, n = 7). PM (1×10^6^/ml) were cultured for 1 h with V_H_CDR3, LPS or NC (all 10 µg/ml), in the presence or absence (NS) of mAb to TNF-α (0.5 µg/ml). After incubation, permeabilized cells were reacted with RPE-labelled mAb to TLR-4 and analyzed by FACScan flow cytometry. Values represent the percentage of positive cells (**B**) *, *P*<0.05 (V_H_CDR3 plus mAb to TNF-α treated *vs* V_H_CDR3 treated cells, n = 7). PM (3×10^6^/ml) were cultured for 1 h as above described. After incubation, cell lysates were subjected to Western blotting. Membranes were incubated with Abs to TLR-4 and actin. TLR-4 production was normalized against actin (**C**) *, *P*<0.05 (V_H_CDR3 plus mAb to TNF-α treated *vs* V_H_CDR3 treated cells, n = 5). For testing the expression level of TLR-4 gene, PM (1×10^6^/ml) were cultured for 1 h as above described. After incubation, total RNA was isolated and analyzed for mRNA expression with RT-PCR. Transcript copy numbers were determined by qPCR using cDNA as a template. Copy numbers were normalized against the copy number of the GADPH gene (**D**). *, *P*<0.05 (V_H_CDR3 plus mAb to TNF-α treated *vs* V_H_CDR3 treated cells, n = 5). Error bars, s.e.m.

To confirm the direct involvement of TNF-α in the enhanced expression of TLR-4 in PM, the production of this cytokine by these cells in the presence or absence of V_H_CDR3 was evaluated. A significant increase of TNF-α production was observed after 1 h of stimulation with V_H_CDR3 (Fig. 6A). Furthermore, we tested the expression of TLR-4 in PM stimulated for 1 h with V_H_CDR3 in the presence or absence of Ab to TNF-α. As reported in Figure 6B, mAb to TNF-α completely abrogated the upregulation of TLR-4 expression induced by V_H_CDR3. To further understand whether the observed downregulation of TLR-4 expression was due to TLR-4 internalization, the TLR-4 protein level was tested by immunoblot analysis. The results reported in Figure 6C showed that there is a strong decrease of TLR-4 protein level in cells treated with anti-TNF-α mAb respect to untreated counterpart. These results seem to be a direct consequence of gene transcription inhibition, as evidenced in Figure 6D.

### V_H_CDR3 Positively Influences the Course of Systemic Candidiasis

Given that V_H_CDR3 is able to induce a state of activation in PM, we tested whether this condition could influence the course of infection in a mouse experimental model of systemic candidiasis, despite the proven non-candidacidal properties of the peptide. Mice were infected intravenously with the opportunistic fungus *C. albicans* and treated with mouse V_H_CDR3 or V_L_CDR3 (used as a negative control) intraperitoneally 4 h before, and 1 and 2 days after infection [18]. Animal survival and fungal burden in kidneys were evaluated in different groups of mice.

**Figure 7:**
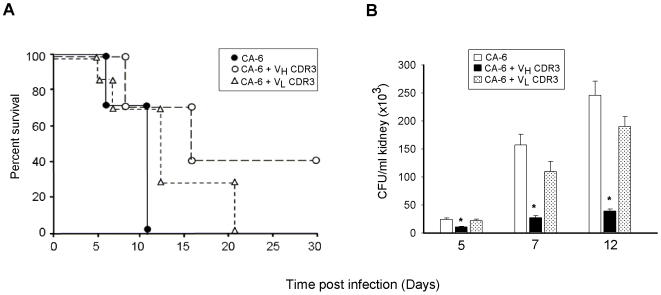
Percent survival and determination of fungal clearance from kidneys of Balb/c mice challenged with *C. albicans (CA-6)* and treated with V_H_CDR3 or V_L_CDR3. Percent survival of infected mice was evaluated according to Mantel-Cox Log rank test and the difference among experimental groups resulted significant (*P*<0.05, n = 7) (**A**). CFU recovery from the kidneys of mice was determined 5, 7 and 12 days after fungal infection (**B**). *, *P*<0.05 (V_H_CDR3 treated *vs* V_H_CDR3 untreated mice, n = 7). Error bars, s.e.m.

Results, reported in Figure 7A, showed a significant increase in survival for infected mice treated with V_H_CDR3 as compared to infected mice untreated or treated with the non immunomodulatory V_L_CDR3. In the same experimental conditions, CFU recovery from kidneys showed an impressive decrease when mice infected with *C. albicans* were treated with V_H_CDR3 as compared to negative control mice (Fig. 7B).

## Discussion

It has been documented that isolated CDR sequences, especially CDR H3, called micro-Abs may show the same binding properties and biological functions displayed by the native Ab [35], [36]. Recently, it has been reported that, independently from the specificity of the native Ab for a given Ag, CDRs other than H3 may frequently display differential *in vitro*, *ex vivo* and/or *in vivo* antimicrobial (*C. albicans*), antiviral (HIV-1), and antitumor (melanoma) activities in a way reminiscent of molecules of early innate immunity [22].

As a proof of concept, here we demonstrate that a synthetic peptide with sequence identical to V_H_CDR3 of a mouse IgM mAb specific for difucosyl human blood group A substance (MoA) could display a potent immunomodulatory activity thus exerting a therapeutic effect against systemic candidiasis without possessing direct candidacidal properties. Significantly the amino acid residue at position 5 (N) proved to be functionally critical for the immunostimulatory properties of V_H_CDR3, as its substitution by alanine abrogated TNF-α production. One possible scenario suggested by these data is that selected short sequences representative of the CDRs of Abs could be strongly involved in inflammatory responses and, as a consequence, in chronic inflammatory processes. V_H_CDR3 peptide is able, indeed, to stimulate PM to produce TNF-α, and this could be instrumental in inducing inflammation. As a matter of fact, TNF-α is considered a classical cytokine of chronic inflammatory disease [37].

Macrophages perform a central task in both the innate and adaptive immune systems. The life and function of these cells are characterized by significant functional versatility. Macrophages ingest foreign materials, present Ags to T lymphocytes in association with the MHC, and can kill microbes and tumor cells upon activation by cytokines and/or T cells [38]. In addition, they eliminate damaged or apoptotic cells. Conversely, macrophages can also release copious amounts of toxic metabolites that can promote tissue damage during antimicrobial defence responses [39].

Our evidence reports that PM very rapidly take up the V_H_CDR3 peptide, and 18 h post treatment this peptide is still associated to the cells. It is possible that V_H_CDR3 could be continuously internalized and degraded within 18 h; alternatively, the peptide could be retained by cells for 18 h and subsequently degraded or expelled.

**Figure 8:**
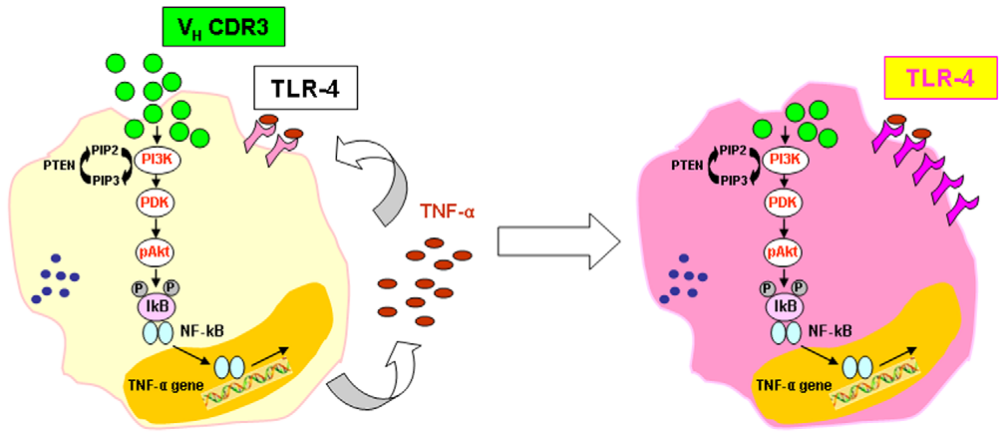
Mechanism of TLR-4 upregulation induced in PM by V_H_CDR3.

PI3K has been linked to an extraordinarily diverse group of cellular functions, including cell growth, proliferation, differentiation, motility, survival and intracellular trafficking. Many of these functions relate to the ability of PI3K to activate Akt [28]. The interaction of V_H_CDR3 with PM induces Akt activation that finally leads to phosphorylation of IkBα , with consequent translocation of NFkB into the nucleus. These molecular events are responsible for cellular activation and subsequent transcription of proinflammatory cytokine genes such as TNF-α. Indeed, this pathway of activation is also confirmed by the inhibition of TNF-α production after blocking the specific Akt signalling pathway. Similarly, involvement of p38 MAPK activation was detected using a specific inhibitor of this pathway. As a matter of fact, TNF-α mRNA was detected 1 h post stimulation with V_H_CDR3, suggesting that the signal transduction pathway from Akt leads to cytokine gene expression, as depicted in Figure 8.

Given that TNF-α is believed to be a positive regulator of TLR-4 expression [31], and that the ability of cells to respond to several microbial motifs depends on TLR-4 expression, we found that, in our experimental system, V_H_CDR3 up-regulates TLR-4. The stimulation of TLR-4 leads to cellular activation [40], and this effect could reinforce the capacity of the peptide to induce inflammatory responses. Moreover, when considering that TLR-4 up-regulation is completely blocked by neutralizing TNF-α, one could posit that the overexpression of TLR-4 is secondary and dependent on TNF-α production.

Convincing arguments point to the key role of TLR-4 in microbial antigen recognition in relation to its protective response [40]. In particular, the antigenic structures of the opportunistic fungus *C. albicans* are recognized by TLR-4 [41]. In our experimental system, a significant increase in survival and a drastic decrease in fungal growth in the kidney, the target organ for *C. albicans*, was surprising, given that V_H_CDR3 is ineffective against *C. albicans* cells *in vitro*. A possible explanation for this could be that natural immune cells are activated by V_H_CDR3 treatment and more prone to ingest and kill *C. albicans*. Additionally, increased TLR-4 expression on PM could facilitate *C. albicans* recognition with consequent more prompt and efficient immune response. The rapid clearance of *C. albicans* observed *in vivo* is particularly relevant, given that V_H_CDR3 does not show any direct candidacidal activity. A simple peptide derived from a mAb specific for difucosyl human blood group A substance, is endowed with potent immunoregulatory effects that are intrinsically able to control the course of a microbial infection.

Whether a proteolytic release of modulatory fragments may physiologically occur beyond the half life of immunoglobulins is an intriguing hypothesis that would account for the apparent redundancy in their production. Nature may have provided extrinsic activities to peptides integrated in evolutionary molecules such as Abs in a way reminescent of human cationic peptides that play an innate immune regulatory role in host defence [24].

Different studies on the mammalian immune system have revealed important interrelationships between adaptive and innate immune response [42]. Preliminary studies have shown that even peptides putatively deriving from the proteolysis of the constant regions of immunoglobulins by physiological enzymes may display immunoregulatory activities on immune cells (unpublished data). Overall our findings suggest that Ab-derived peptides may act likewise effectors of the innate immune response opening a new scenario about their interplay with the cellular immune response.

## Materials and Methods

### Cell Culture Media

RPMI-1640 with L-glutamine and FCS were obtained from Gibco BRL (Paisley, Scotland). Thioglycollate medium was purchased from Difco (BD Biosciences, Franklin Lakes, NJ). All reagents and media were negative for endotoxin, as assessed by *Limulus* amebocyte lysate assay (Sigma Chemical Co., St Louis, MO).

### Microorganism

The origin and characteristics of the highly virulent *C. albicans* strain (CA-6) used in this study have been previously described [43]. The culture was maintained by serial passages on Sabouraud agar (BioMérieux, Lyon, France). The yeast cells were harvested by suspending a single colony in saline, washed twice, counted in a hemocytometer and adjusted to the desired concentration.

### CDRs, Scramble and Negative Control Peptides

The CDRs of human mAb HuA (IgM) and mouse mAb MoA (IgM) specific for the difucosyl human blood group A substance, were chemically synthesized on the basis of the previously described sequences of V_H_ and V_L_ chain [21]. CDRs sequences are listed in Table 1. An irrelevant synthetic decapeptide (MSTAVSKCAT), previously proven to be devoid of either candidacidal or immunomodulatory activity, was used as a negative control (NC) [8], [16]. In selected experiments, a scramble peptide (SP) (YYWLQGGFAN) and nine V_H_CDR3 alanine-substituted derivatives were used for control of specificity and evaluation of key recognition elements of V_H_CDR3.

**Table 1:** CDRs sequences.

CDR	mAb HuA	mAb MoA
V_H_CDR1	SYTFH	SYWIN
V_H_CDR2	VLAYDGSYQHYADSVKG	DIYPGSGITNYNEKFKS
V_H_CDR3	GQTTVTKIDEDY	GQYGNLWFAY
V_L_CDR1	RASQSVSSYLA	RASQDINNYLN
V_L_CDR2	DASNRAT	YTSRLHS
V_L_CDR3	QQRSNWPRS	QQGNTLPWT

### In Vitro Candidacidal Assay of Synthetic mAb MoA CDRs

The *in vitro* activity against CA-6 cells of mAb MoA synthetic CDRs at the concentration of 100 µg/ml was evaluated by a conventional Colony Forming Unit (CFU) assay as described elsewhere [8].

### Mice

Female, 8–10 weeks old, inbred Balb/c mice were obtained from Harlan Nossan Laboratories (Milan, Italy) and housed at the Animal Facilities of the University of Perugia, Perugia, Italy. Procedures involving animals and their care were conducted in conformity with national and international laws and policies.

### Cell Separation

DC and CD4^+^ T cells were separated from spleens of inbred Balb/c mice using N-418 or L3T4 mAb-conjugated MicroBeads (Miltenyi Biotec, Bergisch Gladbach, Germany), and magnetic separation was performed according to the manufacturer's instruction. Peritoneal murine neutrophils (PMN) and macrophages (PM) were collected 18 h or 4 days, respectively, after the intraperitoneal injection of 1 ml endotoxin-free 10% thioglycolate solution (Difco).

### Cytokine Production

Unfractionated spleen cells (10×10^6^/ml), recovered from Balb/c mice, were cultured in the presence or absence of mAb HuA and mAb MoA CDR peptides, LPS or NC (all 10 µg/ml) for 18 h in RPMI-1640 plus 10% FCS (complete medium) at 37°C and 5% CO_2_. After incubation, the supernatants were collected and tested for TNF-α and IL-6 levels by specific ELISA assays (Biosource, Camarillo, CA, USA). In selected experiments, PM (5×10^6^/ml) were cultured as above described. After incubation, the supernatants were collected and tested for TNF-α and IL-6 levels by specific ELISA assays (Biosource). PM (5×10^6^/ml) were cultured in the presence or absence of mouse V_H_CDR3, LPS, NC or SP (all at 10 µg/ml) for 1 or 18 h in complete medium at 37°C and 5% CO_2_. PM (5×10^6^/ml) were cultured as above described in the presence or absence of wortmannin (4 nM) or SB203580 (0,2 µM) (both from Sigma). After incubation, the supernatants were collected and tested for TNF-α level by specific ELISA assays (Biosource). In selected experiments, PM (5×10^6^/ml) were cultured in the presence or absence of V_H_CDR3, LPS, NC, SP and nine V_H_CDR3 alanine-substituted derivatives (all at 10 µg/ml) for 18 h in complete medium at 37°C and 5% CO_2_. After incubation, the supernatants were collected and tested for TNF-α level by specific ELISA assays (Biosource). PMN (5×10^6^/ml) were cultured in the presence or absence of mAb MoA CDR peptides, LPS or NC (all 10 µg/ml) for 18 h in complete medium at 37°C and 5% CO_2_. After incubation, the supernatants were collected and tested for TNF-α and IL-6 levels by specific ELISA assays (Biosource). Cytokine titers were calculated by reference to standard curves, constructed with known amounts of recombinant cytokines.

### Flow Cytometry Analysis

DC, PM, PMN and CD4^+^ T cells (all 1×10^6^/ml) were incubated for 1 h with biotin-labelled mouse V_H_CDR3 (b-V_H_CDR3) or biotin-labelled NC (b-NC) (both 10 µg/ml) in complete medium at 37°C and 5% CO_2_. After incubation, cells were fixed with 4% formalin for 5 min at room temperature (RT), permeabilized with 0.1% saponin, washed with 0.1% saponin and incubated for 20 min on ice with FITC-labelled mAb to biotin (1 µl/test; Mouse IgG1 isotype) (Miltenyi Biotec), washed with 0.1% saponin and analyzed by FACScan flow cytometry (BD Biosciences). Data are reported as percentage of positive cells. In selected experiments, PM (1×10^6^/ml) were incubated for different times with b-V_H_CDR3 or b-NC (both 10 µg/ml) in complete medium at 37°C and 5% CO_2_. After incubation, cells were fixed, permeabilized and washed as above and incubated for 20 min on ice with FITC-labelled mAb to biotin, washed with 0.1% saponin and analyzed by FACScan flow cytometry (BD Biosciences). Data are reported as mean fluorescence intensity (MFI) or percentage of positive cells. For evaluation of TLR-4 expression, PM were incubated for 1 h with mouse V_H_CDR3, LPS or NC (all 10 µg/ml) in complete medium at 37°C and 5% CO_2_. After incubation, cells were fixed, permeabilized and washed as above and incubated for 20 min with R-phycoerythrin (RPE)-labelled mAb to TLR-4 (0.5 µg/10^6^ cells, Rat IgG2a isotype) (Chemicon Int., Temecula, CA). The cells were then washed twice with 0.1% saponin and analyzed by FACScan flow cytometry. Results shown are from one representative experiment out of five with similar results. In selected experiments, PM (1×10^6^/ml) were cultured for 1 h with mouse V_H_CDR3, LPS or NC (all 10 µg/ml) in the presence or absence of mAb to TNF-α (0.5 µg/ml; Armenian Hamster IgG isotype) (BioLegend, San Diego, CA). After incubation, cells were fixed, permeabilized, washed and incubated for 20 min with RPE-labelled mAb to TLR-4. The cells were then washed twice with 0.1% saponin and analyzed by FACScan flow cytometry. Data are expressed as percentage of positive cells. NC staining of cells with irrelevant Abs were used to obtain background fluorescence values.

### Fluorescence Microscopy

In selected experiments, PM were incubated for 1 h with b-V_H_CDR3 or b-NC (both 10 µg/ml) in complete medium at 37°C and 5% CO_2_. After incubation, cells were reacted with FITC-labelled mAb to biotin in the presence of Evans' Blue (StemCell Technologies Inc., Milan, Italy) as a counterstain, and subsequently examined under fluorescent light microscopy (Carl Zeiss, Jena, Germany).

### RNA Extraction

PM (1×10^6^/ml) were incubated for different times in the presence or absence of mouse V_H_CDR3, LPS or NC (all 10 µg/ml) in complete medium at 37°C and 5% CO_2_. In selected experiments PM (1×10^6^/ml) were cultured for 1 h with mouse V_H_CDR3, LPS or NC (all 10 µg/ml) in the presence or absence of mAb to TNF-α (0.5 µg/ml; Armenian Hamster IgG isotype) (BioLegend). After incubation, cells were lysed using Trizol reagent (Invitrogen, Carlsbad, CA), and total RNA was extracted. The reverse transcriptase (RT) reaction was performed using Moloney murine leukaemia virus reverse transcriptase (M-MLV RT), as described in the manufacturer's instructions (Invitrogen).

### Generation of Standards for Real-Time PCR

TNF-α, TLR-4, and GADPH clones for use as standards were prepared by PCR from cDNA derived from PM and cloned into p-Drive vector (Qiagen, S.p.A., Milano, Italy). These were verified by sequence analysis. Plasmid DNA was diluted 10-fold and the starting concentration for the dilution series was 10^8^ gene copies/µl.

### Real-Time RT-PCR (Quantitative RT-PCR)

For each target gene, primers were selected using the Beacon Designer software (Bio-Rad, Hercules, CA). All primers are listed in Table 2. Real-time RT-PCR (quantitative RT-PCR) was performed in 96-well PCR plates using the SYBR green (all from BioRad). For real-time PCR reaction 100 ng/µl of reverse-transcribed RNA was used and cDNA was normalized according to GADPH as an internal NC gene. Amplification conditions were the same for TNF-α, TLR-4 and GADPH mRNAs assayed: 3 min at 95°C, 40 cycles of 10 s at 95°C and 30 s at 62°C. For the Melt Curve, the amplification condition was: 1 min at 50°C and 90 cycles of 10 s at 50°C. The experiments were performed using the iCycler IQ Multicolor Real-time PCR Detection System (BioRad).

**Table 2:** Primer sequences used for quantitative RT-PCR.

Gene	Forward primer (5′-3′)	Reverse primer (5′-3′)	Fragment size (bp)
GADPH	GCCTTCCGTGTTCCTACCC	CAGTGGGCCCTCAGATGC	117
TNF-α	CGCTCTTCTGTCTACTGAACTTCG	GATGATCTGAGTGTGAGGGTCTGG	115
TLR-4	CACTGTTCTTCTCCTGCCTGAC	AGGGACTTTGCTGAGTTTCTGATC	104

Primers were designed with the help of Beacon Designer software (Bio-Rad, Hercules, CA, USA) and provided by Invitrogen.

### Western Blotting for Phospo-Akt, Phospo-IkBα and TLR-4

PM (3×10^6^/ml) were incubated for 1 or 3 h in the presence or absence of mouse V_H_CDR3, LPS, NC or SP (all 10 µg/ml) in complete medium at 37°C and 5% CO_2_. In selected experiments PM (3×10^6^/ml) were incubated for 1 h in the presence or absence of wortmannin (4 nM) (Sigma) and mouse V_H_CDR3, LPS or NC (all at 10 µg/ml) in complete medium at 37°C and 5% CO_2_. After incubation, cells were washed and lysated with Mammalian protein extraction reagent (M-PER) in the presence of protease and phosphatase inhibitors (all from Pierce, Rockford, IL). Protein concentration was determined with a BCA protein Assay Reagent kit (Pierce). The lysates (30 µg of each sample) were separated by sodium dodecyl-sulfate-10% PAGE, transferred to a nitrocellulose membrane (Pierce) for 1 h at 100 V in a blotting system (Bio-Rad) for Western Blot analysis, and the membranes were incubated over-night with rabbit polyclonal Abs to phospho-Akt (pAkt; dilution 1/1000), phospho-IkBα (pIkBα; dilution 1/1000) (all from Cell Signalling Technology, Beverly, MA) and rabbit polyclonal Ab to TLR-4 (dilution 1/200) (Santa Cruz Biotechnology Inc.) in blocking buffer. Detection was achieved with the appropriate secondary Abs coupled to HRP, followed by ChemiLucent Trial Kit (Chemicon Int.). Immunoblotting with rabbit polyclonal Abs to Akt, IkBα and actin (all dilution 1/1000) (all from Cell Signalling Technology) were used as internal loading controls to ensure equivalent amounts of protein in each lane. Immunoreactive bands were visualized and quantified by Chemidoc Instrument (Bio-Rad). In particular, a quantitative analysis of the region of interest drawn around the outer edge of the band, was performed based on the guidelines for the use of the Chemidoc Instrument (Bio-Rad). This analysis is defined as “volumetric” because the software of Chemidoc Instrument transforms each pixel that constitute a band in a parallelogram as higher as bigger his intensity. All the parallelograms that form a band create one unique three-dimensional solid structure on which the volume is calculated. In this way each pixel has given the right contribution to the final volumetric analysis. Moreover, for the correct analysis of the bands the background from all the bands with Global methods based on the guidelines of the Chemidoc Instrument software was excluded.

### Evaluation of the Therapeutic Activity of Selected mAb MoA CDRs against Systemic Candidiasis

Groups of 7 Balb/c mice were infected intravenously with 5×10^5^ or 7.5×10^5^ CA-6 cells suspended in 0.5 ml saline for evaluation of fungal clearance or mice survival, respectively.

Infected animals were given 200 µg of mouse V_L_CDR3 or V_H_CDR3 peptides intraperitoneally 4 h before infection, and 100 µg 1 and 2 days after infection for a total of 400 µg/mouse [18]. Untreated animals served as a NC. Quantification of fungal growth at 5, 7, and 12 days after infection was assessed by plating serial dilutions of kidney homogenates onto Sabouraud agar. Animal survival was monitored for 30 days after infection.

### Statistical Analysis

Data are reported as the mean ± s.e.m. from 5–7 separate experiments and the Student's *t* test was used to determine the statistical significance of differences between experimental groups. The Kaplan-Meier and Log rank tests were applied to survival data. A value of *P*<0.05 was considered significant.
